# Evaluating a new voluntary occupational health and safety management system program in the context of a pandemic

**DOI:** 10.5271/sjweh.4285

**Published:** 2026-07-01

**Authors:** Laksika B Sivaraj, Robert A Macpherson, Christopher B McLeod

**Affiliations:** 1 Partnership for Work, Health and Safety, School of Population and Public Health, University of British Columbia, Vancouver, British Columbia, Canada.

**Keywords:** Canada, COVID-19, difference-in-difference, intervention, workplace injury

## Abstract

**Objectives:**

Shortly before the COVID-19 pandemic, the Workplace Safety and Insurance Board of Ontario, Canada, launched the Health and Safety Excellence program (HSEp), a new voluntary occupational health and safety management system (OHSMS) program. This study conducted a prospective evaluation for the impact of HSEp participation on injury rate reduction during an ongoing pandemic.

**Methods:**

A difference-in-differences study design with a hybrid matching approach was used to evaluate HSEp’s effectiveness in reducing lost-time injury rates with and without COVID-19 claims among participating firms compared with similar non-participating firms. The analysis was stratified by enrollment cohort, industry, firm size, and prior OHSMS experience.

**Results:**

A total of 1680 matched HSEp firms were followed for 48 months. Some evidence of an effect on lost-time injury rates was observed, particularly after excluding COVID-19 claims. Non-COVID-19 lost-time rate reductions were pronounced among firms that enrolled later [incidence rate ratio (IRR) 0.89, 95% confidence interval (CI) 0.82–0.98], manufacturing firms (IRR 0.81, CI 0.68–0.96), larger firms (IRR 0.92, CI 0.85–0.99), and firms that had progressed through the program (IRR 0.91, CI 0.83–0.98). However, the reductions were not apparent among healthcare and construction companies or smaller firms.

**Conclusions:**

Although the evidence of overall injury rate reductions is limited, emerging evidence suggests that participating in HSEp is associated with reductions in non-COVID-19 injuries in certain contexts. These findings suggest that the OHSMS program performance was affected by the pandemic, and accounting for this was crucial in assessing an intervention effect.

Effective workplace injury management ensures occupational health and safety (OHS) and promotes healthier work environments. Since the late 20^th^ century, OHS management systems (OHSMS) have become a principal approach through which OHS regulators engage with firms to improve workplace safety (1). Despite efforts to improve OHS, globally approximately 395 million non-fatal occupational injuries and 3 million fatalities are reported annually, and challenges in motivating firms to adopt OHS management practices persist (2). To mitigate workplace injuries and diseases and increase uptake of OHS practices, new OHSMS continue to be introduced and implemented in Canada and internationally (3, 4).

The coronavirus disease (COVID-19) pandemic had a significant impact on businesses worldwide. Its effects extended beyond the economic environment to affect organizational performance, work structures, and most importantly, employee health and safety (5, 6). As the pandemic progressed, public health interventions, including industry shutdowns, social distancing requirements, and mask and vaccine mandates, significantly altered work environments. Organizations that set the standards and validate OHSMS, as well as the employers implementing and managing them, faced significant challenges in maintaining safety standards, as these systems were not originally designed to address pandemic-specific risks or the operational disruptions associated with large-scale public health emergencies.

Despite the widespread implementation of OHSMS, there is a lack of literature on evaluating the performance of OHSMS implemented during the pandemic. Even existing studies on the effectiveness of OHSMS interventions in reducing pre-pandemic workplace injuries present mixed findings (1, 7–15). While some report reductions in injury rates in specific contexts, others raise concerns about the overall impact of these programs. However, due to methodological limitations in many of these evaluations, such as insufficient adjustment for the heterogeneity of participating firms or lack of consideration for variation in program design, these mixed results may be attributed more to the study design limitations rather than the underlying OHSMS framework itself. Beyond these gaps, it remains unclear how the pandemic-related claims may have further influenced the performance evaluation of OHSMS in terms of workplace injury reduction.

In November 2019, just before the onset of the pandemic, the Workplace Safety and Insurance Board (WSIB) of Ontario, Canada, launched the Health and Safety Excellence program (HSEp) (16). The goal was to help businesses improve workplace safety by encouraging investment in effective health and safety practices. However, soon after the launch, the pandemic began to significantly impact businesses across Ontario, and consequently enrollment and participation in the HSEp was effected. Because the program was not originally designed to address pandemic-related risks, its effectiveness in this new context was uncertain, as was that of other OHSMS. This study examines the program’s effectiveness on injury reduction in an on-going pandemic through a quasi-experimental study design. The aim of this study was to assess HSEp’s effectiveness in reducing the rate of workplace lost-time injuries with and without COVID-19 injuries over time.

## Methods

### The Ontario Health and Safety Excellence Program

In Ontario, Canada, the WSIB oversees the provision of wage-loss benefits, medical coverage, and return-to-work support for individuals affected by work-related injuries or illnesses. Funded by premiums paid by Ontario businesses, the WSIB offers no-fault collective liability insurance and delivers industry-specific health and safety resources to over five million workers across more than 300 000 workplaces (17). These claim-level workers’ compensation data, collected for administrative purposes, contain information on workers’ injury claims submitted by firms. To enroll in the HSEp, firms must first register with a WSIB-approved program provider and work towards completing action plans. An action plan consists of program topics, which firms have up to 12 months to complete and validate. Successful completion of an action plan indicates that the firm has implemented the required safety measures related to the selected action plan topics. In this study, action plan completion serves as an indicator of program progression. The HSEp is currently comprised of 41 OHS topics (36 topics when launched) distributed across three program levels: foundation, intermediate, and advanced. While the program was not originally intended to address pandemics, several changes were made in response to COVID-19 in 2020, including introducing new safety topics related to the pandemic and increasing flexibility to modify selected topics within an approved action plan. Firms that complete all topics in a particular program level are rewarded a badge of recognition and a workers’ compensation premium rebate.

### Study design and data

This study employed a retrospective cohort design using a matched difference-in-differences (DiD) approach, utilizing WSIB HSEp, firm and claim-level data. Data for firms registered with WSIB from 2016 to 2024 were used. These data consisted of firm characteristics, including firm start and end dates, classification unit, industry sector, annual full-time equivalent (FTE), region, and schedule type (self-insured or not). Data were provided at the firm sub-account or classification unit level and subsequently aggregated to the account level. Based on each account’s primary industry classification, the annual account-level FTE estimates were then disaggregated to the monthly level using industry-level monthly employee data from Statistics Canada (18). Firms’ participation in HSEp was available through HSEp portal data from the program start date (November 2019) to the end of 2024. In addition, information on firms’ participation in other safety programs [eg, Certificate of Recognition (COR); Safety Groups program – discontinued; Supporting Ontario’s Safe Employers) was also accessed (19, 20). Compensation claim data from 2016 to 2024 were used, including injury date, claim type, and injury type and description.

### Study cohorts

The study comprised two cohorts: Year 1 participants included all firms registered with the WSIB in 2019, and Year 2 participants included all firms registered in 2020. Enrollment in the HSEp and the time of enrollment were based on the program enrollment date. Year 1 participating cases included all firms that enrolled between HSEp’s initiation on 1 November 2019 and 31 March 2020. Year 2 participating cases included all firms that enrolled between 1 April 2020 and 31 March 2021. Eligible controls for each cohort were defined as firms that did not enroll in the HSEp at any point in time as of study end (end of 2024). Eligibility for each cohort was determined using a set of inclusion/exclusion criteria, based on firm characteristics, enrollment in other safety programs, and injury claims (supplementary material, www.sjweh.fi/article/4285, table S1).

### Study timeline

**Figure 1:**
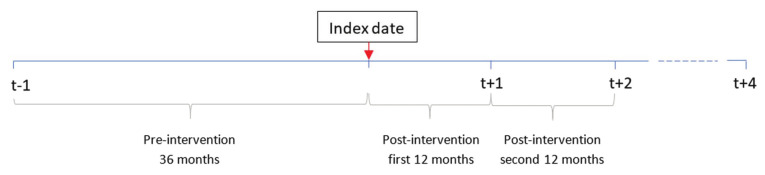
Illustration of the analysis timeline.

All firms were assigned an index date to separate pre- and post-intervention time periods. For cases, HSEp enrollment date and index date were synonymous. For controls, an index date was randomly assigned from a list of all case enrollment dates observed in each cohort. This assignment occurred before matching to allow the creation of matching variables (eg, pre-intervention lost-time claims indicator). Further, these predefined pre- and post-intervention periods were also used to determine firms’ eligibility for matching, maximizing retention of the cases while ensuring the comparable observation windows within matched pairs in a given year. The pre-intervention period for cases and controls was defined as 36 months preceding the calendar month of their index date. The post-intervention period was up to four 12-month periods following the index month (figure 1).

### Hybrid matching

To address selection bias, comparable control groups for each cohort were identified through a hybrid matching approach that combined exact matching with nearest-neighbor propensity score (21). A one-to-one matching without replacement was performed, with each case matched to a single control within the same cohort. Exact matching was performed based on the baseline firm size, one-digit level North American Industry Classification System (NAICS) industry group, Safety Groups experience, pre-intervention lost-time claim history, and COR status. Nearest-neighbor matching was conducted based on the three-digit NAICS sub-sector codes. Matched pairs were pruned where matching resulted in NAICS sub-sectors with substantial differences. For each cohort, all baseline characteristics were based on their first calendar year, so that the baseline characteristics were measured prospectively. Detailed descriptions of these variables and baseline lost-time injury claim rates for pre- and post-matching samples are listed in supplementary tables S2 and S3, respectively.

### Outcome measures

The study outcomes were based on WSIB-approved lost-time injury claims (22). These are claims reported due to work-related injury or illness, where the injured worker is medically unable to perform their regular job duties and has lost time from work beyond the day of the incident, resulting in a loss of earnings. To assess the pandemic impact on the workplace injury rates, lost-time claims excluding COVID-19 (non-COVID-19 lost-time injuries) were analyzed. COVID-19 claims were identified using the nature of injury code 14360 for “COVID-19 novel coronavirus”. For each claim type, monthly claim counts were aggregated at the firm level. The firm-level monthly injury counts derived from each claim type served as the primary outcomes in this study.

### Statistical analysis

Each outcome was analyzed separately using population-averaged (generalized estimating equation) negative binomial regression models with an independent correlation matrix and offset of log monthly FTE. The analysis focused on changes in the collective post-intervention period as well as each 12-month time point compared to the 36-month pre-intervention period (figure 1). The covariates comprised calendar year (2016–2024), month, firm region, firm size, and NAICS sector. COR firms were excluded from the final sample (82 and 50 matched pairs from Year 1 and Year 2 participants, respectively) as previous research has shown how COR participation is associated with reductions in the injury outcomes (7). Each outcome was examined by each cohort overall and then stratified by industry sector (construction, manufacturing, healthcare and social assistance, and services), firm size (small/medium <100 and large ≥100 FTE), and prior Safety Groups experience (some versus none).

### Sensitivity analysis

Given that healthcare sector firms operated and were affected differently during the pandemic compared to other sectors, a post-hoc analysis was conducted to assess the overall effect, focusing on non-healthcare sectors. Here, the primary analyses for lost-time injuries were repeated after excluding healthcare and social assistance firms. Further, to assess the effect of progression in the HSEp, participating firms that completed at least one action plan and those that did not were analyzed separately, along with their corresponding matched controls. This analysis focused on both lost-time injuries and non-COVID-19 lost-time injuries.

All analyses were performed using SAS 9.4 version, and the significance of the findings was assessed using a 95% confidence interval (CI). The preferred regression models were chosen based on the lowest quasi-likelihood information criterion (QIC) value (23).

## Results

The largest share of HSEp firms in each cohort operated in the construction/utilities/mining/oil & gas sectors, followed by manufacturing (table 1). The firm size distribution was more uniform, with 53% being small or medium-sized firms. Year 1 participants had a larger share of firms with Safety Groups experience (63%) than Year 2 participants (37%). Following the exclusion of COR firms, there were 974 HSEp firms in Year 1 participants and 706 in Year 2 participants. Temporal trends show that both the HSEp and non-HSEp firms experienced a decline in non-COVID lost-time claims and FTE during their first year of follow-up for the Year 1 participants, which approximately corresponds to calendar year 2020. The remaining follow-up period for Year 1 participants and the full follow-up for the Year 2 participants were associated with gradually increasing claim counts and FTE in general. However, overall lost-time claims increased over time since the baseline as a result of COVID-19 related claims.

**Table 1:** Firm characteristics for the pre- and post-match samples. [CAD=Canadian dollars; FTE=full-time equivalent employees. NAICS=North American Industry Classification System; SD=standard deviation].

		Year 1 participants						Year 2 participants				
		Pre-match N=1302			Post-match N=1056			Pre-match N=779			Post-match N=756	
		N (%)	Mean (SD)		N (%)	Mean (SD)		N (%)	Mean (SD)		N (%)	Mean (SD)
NAICS												
	Construction, utilities, mining, oil & gas	414 (31.8)			350 (33.2)			217 (27.9)			211 (27.9)	
	Manufacturing	322 (24.7)			291 (27.6)			201 (25.8)			201 (26.6)	
	Education, healthcare, social assistance	205 (15.7)			117 (11.1)			92 (11.8)			88 (11.6)	
	Administraton, finance, information and culture, professional, tech	122 (9.4)			82 (7.8)			61 (7.8)			61 (8.1)	
	Retail, wholesale, transportation and warehousing	121 (9.3)			121 (11.5)			122 (15.7)			116 (15.4)	
	Public administraton, leisure, hospitality	102 (7.8)			82 (7.8)			62 (5.1)			40 (5.3)	
	Agriculture, forestry, fishing	16 (1.2)			13 (1.2)			24 (3.1)			18 (2.4)	
Firm size (FTE)												
	<20	185 (14.2)			161 (15.3)			146 (18.7)			144 (19.1)	
	20–<50	253 (19.4)			204 (19.3)			133 (17.1)			131 (17.3)	
	50–<100	253 (19.4)			206 (19.5)			130 (16.7)			130 (17.2)	
	100–<500	446 (34.3)			362 (34.3)			273 (35)			263 (34.8)	
	≥500	165 (12.7)			123 (11.7)			97 (12.5)			88 (11.6)	
Safety Groups experience												
	Yes	826 (63.4)			582 (55.1)			292 (37.5)			271 (35.9)	
Region												
	Central Ontario	569 (43.7)			423 (40.1)			295(37.9)			285 (37.7)	
	North, East Ontario	182 (14.0)			161 (15.3)			169 (21.7)			162 (21.4)	
	West Ontario	486 (37.3)			425 (40.3)			260 (33.4)			255 (33.7)	
	Outside Ontario	65 (5.0)			47 (4.5)			55 (7.1)			54 (7.1)	
Insurable earnings (CAD x10^6^)			16.6 (54.3)			17.4 (59.7)			19.4 (69.2)			18.1 (64.3)
Individual premium (CAD x10^4^)			28.8 (75.1)			28.7 (80.0)			24.1 (65.2)			22.6 (57.2)

### Cohort-specific findings

According to the generalized models, Year 1 and Year 2 participants showed no statistically significant intervention effects on lost-time injury rates over time (table 2). However, after excluding COVID-19 claims, 9% (95% CI <1–16%) and 11% (95% CI 2–18%) overall reductions were observed among Year 1 and Year 2 HSEp participants relative to the observed changes among non-participants, respectively. The yearly effect estimates became more precise and consistent throughout the follow-up period. For Year 1 HSEp participants, a statistically significant 15% (95% CI 5–25%) reduction in non-COVID-19 lost-time injuries was observed in the fourth year of follow-up. Further, with the exclusion of COVID-19 claims, Year 1 HSEp participants observed a 5% reduction in year 1, compared with a 23% increase when these claims were included, however, these changes were not statistically significant (P=0.050). For Year 2 HSEp participants, statistically significant injury rate reductions up to 13% (95% CI 1–24%) were observed during the follow-up for non-COVID-19 lost-time injuries.

**Table 2:** Intervention effects of Health and Safety Excellence program (HSEp) participation on injury claim rates, by cohort. Estimates derived from matched adjusted population-averaged negative binomial regression models. [COM=combined; IRR=incidence rate ratio; CI=confidence interval.]

	Follow-up	Lost-time claim rates			Non-COVID lost-time claim rates	
		IRR (95% CI)	P-value		IRR (95% CI)	P-value
Year 1 participants	COM	1.03 (0.90–1.17)	0.676		0.91 (0.84–1.00)	0.043
	Year 1	1.23 (1.00–1.52)	0.050		0.95 (0.86–1.05)	0.323
	Year 2	0.93 (0.79–1.10)	0.410		0.92 (0.82–1.03)	0.146
	Year 3	1.06 (0.89–1.25)	0.516		0.95 (0.85–1.06)	0.344
	Year 4	0.91 (0.79–1.04)	0.160		0.85 (0.75–0.95)	0.007
Year 2 participants	COM	0.89 (0.76–1.03)	0.124		0.89 (0.82–0.98)	0.017
	Year 1	0.91 (0.72–1.14)	0.417		0.95 (0.85–1.05)	0.321
	Year 2	0.86 (0.73–1.00)	0.057		0.87 (0.77–0.97)	0.013
	Year 3	0.92 (0.79–1.07)	0.290		0.87 (0.76–0.99)	0.038

### Industry-specific findings

Manufacturing firms participating in HSEp indicated the strongest effect with overall significant injury rate reductions of 17% (95% CI 4–28%) for lost-time injuries and 19% (95% CI 4–32%) for non-COVID-19 lost-time injuries (table 3). During follow-up, the intervention effect ranged between 21–27% and 13–26% for lost-time injuries and non-COVID-19 lost-time injuries, respectively. Service sector firms showed some indication of an intervention effect at the end of the follow-up period, though it was not statistically significant for most follow-up years. In general, injury rates were high among the healthcare firms, and no intervention effect was observed for both lost-time and non-COVID-19 lost-time injuries. However, excluding COVID-19 claims resulted in greater precision in the effect estimates. In construction firms, no statistically significant intervention effect was observed for either of the injury outcomes.

**Table 3:** Intervention effects of Health and Safety Excellence program (HSEp) participation on injury rates, by industry sector. Estimates derived from matched adjusted population-averaged negative binomial regression models. [COM=combined; IRR=incidence rate ratio; CI=confidence interval.]

	Follow-up	Lost-time injury rates			Non-COVID lost-time injury rates	
		IRR (95% CI)	P-value		IRR (95% CI)	P-value
Construction	COM	0.97 (0.78–1.20)	0.763		0.91 (0.76–1.09)	0.300
	Year 1	0.84 (0.66–1.07)	0.162		0.84 (0.67–1.06)	0.150
	Year 2	0.98 (0.69–1.39)	0.914		0.87 (0.69–1.08)	0.210
	Year 3	1.15 (0.86–1.56)	0.347		1.04 (0.81–1.33)	0.769
	Year 4	0.92 (0.70–1.19)	0.517		0.90 (0.69–1.17)	0.425
Manufacturing	COM	0.83 (0.72–0.96)	0.011		0.81 (0.68–0.96)	0.018
	Year 1	1.01 (0.78–1.31)	0.926		0.92 (0.79–1.06)	0.236
	Year 2	0.79 (0.65–0.94)	0.010		0.83 (0.71–0.98)	0.023
	Year 3	0.79 (0.68–0.93)	0.004		0.78 (0.67–0.91)	0.002
	Year 4	0.73 (0.62–0.86)	<0.001		0.74 (0.63–0.87)	<0.001
Healthcare	COM	1.13 (0.94–1.37)	0.192		1.11 (0.95–1.29)	0.182
	Year 1	1.19 (0.85–1.68)	0.307		1.03 (0.86–1.23)	0.744
	Year 2	0.96 (0.73–1.26)	0.769		1.07 (0.89–1.28)	0.487
	Year 3	1.24 (0.96–1.60)	0.102		1.12 (0.93–1.35)	0.243
	Year 4	1.41 (1.08–1.84)	0.012		1.22 (1.00–1.49)	0.052
Services	COM	0.92 (0.81–1.05)	0.207		0.91 (0.81–1.02)	0.114
	Year 1	1.01 (0.84–1.21)	0.937		0.96 (0.83–1.11)	0.616
	Year 2	0.94 (0.80–1.10)	0.418		0.89 (0.77–1.04)	0.139
	Year 3	0.92 (0.79–1.07)	0.291		0.95 (0.83–1.10)	0.490
	Year 4	0.82 (0.69–0.98)	0.032		0.84 (0.70–1.01)	0.060

### Firm size-specific findings

Among small and medium-sized HSEp firms, there was some indication of an effect on overall lost-time injuries, but the findings were imprecise (table 4). Excluding COVID-19 claims did not considerably impact small and medium-sized firms. While no notable lost-time rate reductions were observed among large firms participating in HSEp, an 8% (95% CI 1–15%) overall injry rate reduction was observed for non-COVID-19 lost-time injuries. Over time, statistically significant non-COVID-19 lost-time injury rate reductions up to 11% (95% CI 1–20%) were observed among large HSEp participating firms.

### Safety Groups experience-specific findings

HSEp firms with no prior Safety Groups experience indicated some evidence of an intervention effect on lost-time injuries, with rate reductions of 16% (95% CI 1–29%) in follow-up year four (supplementary table S4). A similar effect was observed for non-COVID-19 lost-time injuries, with improved precision in the early follow-up years compared to lost-time injuries. The impact of excluding COVID-19 claims was more apparent among firms with Safety Groups experience, however, no statistically significant effects were observed.

For the preferred models of the main analyses, Type-III test results of the covariates and the model QIC are provided in supplementary table S5.

### Sensitivity analyses findings

The intervention effect on lost-time injuries after excluding healthcare firms largely followed a pattern similar to that observed among lost-time injuries in the pre-exclusion analysis, but with a few significant injury reductions. Intervention effects were observed among firms in both cohorts (up to 25%), large firms (23%), and those with Safety Groups experience (20%) (supplementary table S6). Similarly, there were limited evidence on progression effect on injury rate reduction. In general, an intervention effect of action plan completion on overall lost-time injuries was not observed (supplementary table S7). Although, a statistically significant 9% (95% CI 2–17%) overall rate reduction was observed among non-COVID-19 lost-time injuries for firms that completed an action plan. Further, these firms observed rate reductions in non-COVID-19 lost-time injuries in follow-up year two (IRR 0.89, 95% CI 0.80–0.99) and four (IRR 0.86, 95% CI 0.76–0.98).

## Discussion

### Main findings

The purpose of this study was to determine whether the introduction of the HSEp was associated with reductions in workplace injury rates and assess the degree to which the COVID-19 pandemic-related injuries impacted the findings. Findings from the matched multicohort DiD regression analysis suggest there is some evidence of an effect among firms with specific firm and cohort characteristics that varied based on the injury type and follow-up period. The lost-time injury rate reductions were more pronounced after excluding COVID-19-related claims, suggesting a strong pandemic effect on program effectiveness more generally.

### Interpretation of findings

Comparing the findings of this study with those of prior research is challenging for several reasons. Many OHSMS programs provide certification or formal recognition upon successful completion, and their evaluations are commonly conducted following this point (eg, OHSAS 18001, COR) (11, 12). In contrast, the HSEp is designed differently, offering monetary incentives to firms as they progress through the program, without requiring full completion. As a result, aligning with elements of a developmental evaluation approach, the present evaluation assesses the intervention’s effect during active program participation rather than post-completion (24). Beyond these programmatic differences, the existing literature on the effectiveness of OHSMS is focused on pre-pandemic work environments, while the present study examines pandemic and post-pandemic contexts.

Given that most program evaluations are based on pre-pandemic data, comparisons of their findings with non-COVID-19 injuries in the current study are likely to be more appropriate. Regardless of differences in study design and cohort composition, the current findings align with evaluations of OHSAS 18001 and COR programs, which reported more pronounced reductions in similar yet pre-pandemic injuries during later years of follow-up. In the current study, we observed a 9–11% overall decrease in non-COVID-19 lost-time injury rates among firms that enrolled in HSEp compared to those that did not. Comparable program effects were reported in OHSAS 18001 evaluations conducted in Spain (1.5% reduction), Denmark (15% reduction), and in the United States (20–24% reduction) (10, 12, 13, 25). However, in contrast to the current study, those evaluations focused on overall reductions in work accidents or on different types of injuries, and/or employed different methodological approaches. Additionally, a series of COR program evaluations across various provinces in Canada reported reductions of 12–28% in injuries of a similar nature (7, 8, 11).

Among manufacturing firms, the dynamic analysis indicates a significant reduction of 17–26% in non-COVID-19 lost-time injury rates over the four-year post-intervention period, with an overall reduction of 13%, and no intervention effects were observed in other sectors, including construction. These patterns mirror findings of an OHSAS 18001 evaluation conducted in Spain, where injury reductions were observed in manufacturing firms but not in construction (10). Further, COR program evaluations similarly documented substantial effects among manufacturing firms, along with construction firms (8, 11). These stronger intervention effects among manufacturing firms are likely attributable to this sector being comprised mainly of large firms with stable working environments and greater access to resources, allowing them to implement OHSMS more effectively than sectors such as construction or services.

Furthermore, in the present study, intervention effects on non-COVID-19 lost-time injuries were more apparent among large firms, with an overall rate reduction of 8% and dynamic analysis rate reductions of up to 11%. By comparison, COR evaluations of large firms have reported overall rate reductions in similar injuries of up to 32% (7, 11). Findings for smaller firms in the current evaluation are generally consistent with limited evaluations of programs targeting small enterprises conducted in Canada, such as Small Employer COR, which have similarly found little evidence of effectiveness in reducing comparable injuries (8, 11). Firms that had prior OHS program experience through Safety Groups, did not experience any reduction in injury rates whereas firms with no prior OHS program experience did. Reasons for this may include that despite being newly enrolled in HSEp, these firms already had several aspects of an OHSMS in place whereas other firms did not.

### Pandemic effect

The crude injury claim counts and FTE for both HSEp and non-HSEp firms reflect the pandemic impact on the overall lost-time injury rates, which increased over this period. However, when pandemic-related -injuries are excluded, non-COVID lost-time injury counts decreased in the first follow-up year for Year 1 participants, but later increased over time (for both Year 1 and Year 2 participants). This pattern consistent with the provincial statistics reflecting changes in FTE and injury claim counts (26). Hence the relative reductions observed among HSEp firms during the model fitting are relative to changes in non-HSEp firms and may not represent absolute reductions in injury rates.

Among the analyses that included COVID-19-related injuries, the observed intervention effect appears notably weaker, with wider confidence intervals. For instance, in the manufacturing sector and among large firms, the precision of the effect estimates improved substantially after COVID-19 injuries were excluded. Although the sample sizes remained the same, removing COVID-19 injuries likely reduced variability in the data, leading to lower standard errors and therefore more precise estimates with narrower confidence intervals. This is expected given the high uncertainty and instability in work environments and injury risk and injury claiming during the pandemic. While the program was not initially designed to address the pandemic-related safety challenges, new topics focusing on pandemic management were introduced during the peak of the pandemic. Despite these additions, firms continued to struggle, due in part to varying regulatory requirements, evolving public health policies, and performance standards of their businesses. While some industries were able to adapt through remote or hybrid work arrangements due to the nature of their sector-specific operations, others faced persistent and disproportionate challenges. This was evident in the post-hoc analyses following the exclusion of healthcare firms.

The lack of intervention effect among the healthcare sector, regardless of the exclusion of COVID-19-related injuries, most likely can be explained by the greater burden of the pandemic falling on healthcare workers. During the pandemic, healthcare workers experienced extended shifts, increased exposure to health risks from the virus, and prolonged use of personal protective equipment. As a result, the healthcare sector bore a disproportionate share of the pandemic’s impact, along with an increased likelihood of injuries (27–30). Hence, excluding lost-time claims submitted by this sector provided a clear view of the program’s effectiveness under more typical working conditions.

During the pandemic, many industries faced reductions in onsite employment, due to the changes in the workplace policies, and redundancies. These reductions may have affected non-COVID-19 injury rates in different ways. On the one hand, reduced onsite workforces can affect the implementation and quality control of safety standards resulting in an increase in injuries. On the other hand, due to reduced onsite workforces, firms can experience decrease in general workplace injuries. While our rigorous matching approach may have addressed the employment differences among the treatment and control groups, variations may still have affected our findings over time. Further, pandemic effects on program implementation and progression may also have impacted the effectiveness of the HSEp.

### Progression effect

While the initial intervention effect revealed a weak and inconsistent association between program enrollment and injury rates, a stronger signal of effectiveness emerged among firms that progressed through the program, particularly for non-COVID-19 lost-time injuries. The program’s design suggests that progression, measured by the successful completion of action plans, is more directly comparable to the findings from other program evaluations, rather than the effect of mere enrollment. According to current findings, firms that advanced through the program experienced reductions of up to 14% in non-COVID-19 lost-time injuries. While the observed reductions in non-COVID-19 lost-time injuries among progressing firms may reflect genuine improvements in workplace safety, they may also be due to knowledge transfers related to efficient or proactive claim handling. Selection and progression through the program early on may have been motivated by topics seen relevant to protecting workers from the pandemic. Hence program progression could be correlated with higher incidence of COVID-19-related injuries. Further, more severe injuries that might previously have been reported as lost-time claims may instead have been classified as no-lost-time claims following program progression. A similar trend was observed in the evaluation of the COR program in Alberta, where the reduction in lost-time injuries was potentially driven by increased use of modified work arrangements whereby lost-time claims could then be classified as disabling injury claims (11).

### Strengths and limitations

This study is one of the first, if not the only to evaluate the effectiveness of OHSMS in reducing workplace injury rates during the pandemic. Findings from these evaluations provide insight into how to implement and evaluate OHS program in complex, uncertain circumstances such as a pandemic. This study offers insights into how program maturity may influence effectiveness over time through assessments conducted at multiple post-intervention points. Moreover, this study design enables the alignment of the program modification, policy changes, and pandemic-related disruptions with outcomes observed across specific cohorts and follow-up periods.

The hybrid matching approach employed enabled retention of >87% of the intervention sample while mitigating potential selection bias. The DiD study design allowed for an unbiased estimation of the intervention’s effect under the assumption of parallel trends between intervention and control groups, which was not violated. By using multiple 12-month periods of data, this study applies an extended DiD that evaluates treatment effects across multiple time points to address limitations of a traditional DiD that only compares one pre- and one post-intervention period. Further, the disaggregation of effects associated with overall program participation and program progression enabled comparisons with evaluations of other OHSMS interventions and accounting for structural variations within the program. Finally, the study was designed to account for confounding factors, explicitly the effects of the pandemic, by analyzing injury rates excluding COVID-19-related claims and healthcare firms, thereby contributing to a more refined understanding of how external shocks may affect program performances.

However, unobserved differences may remain between HSEp and non-HSEp firms despite matching. Because HSEp is a voluntary program, firms that proactively enroll may have inherent advantages such as stronger emphasis on safety, greater access to resources, and additional managerial support. These qualities may allow them to be more attentive to both internal and external shocks, enabling them to operate more strategically and efficiently, even during a pandemic. Consequently, the observed injury rate reductions among participating firms may reflect these characteristics, the impact of HSEp itself, or a combination of both.

Further, this study relied on administrative data collected for operational purposes rather than research. As such, these data are subject to potential errors, misclassifications, underreporting, and are sensitive to external disruptions, which can lead to an underestimation of true injury rates. Additionally, due to the nature of administrative data and limitations in disease diagnosis, this study may have underestimated occupational diseases with long latency periods. Furthermore, injury rates were calculated using estimates of FTE at the firm level, derived from reported annual insurable earnings and industry average earnings. This approach may introduce measurement bias into FTE calculations, as the occurrence of workplace injuries themselves can influence insurable earnings. Further, inconsistencies between a firm’s reported industry classification and the industry-level estimates can introduce additional bias into FTE calculations. The conversion of annual FTE into monthly values using employment data from Statistics Canada may also result in an underestimation of FTE, particularly for sectors and firms with unstable labor patterns. Especially, due to the pandemic impact on employment, estimated FTE may be less reliable. Finally, although the analysis accounted for participation in several major OHSMS programs (eg, Safety Groups), due to the data limitations, consideration of all relevant safety programs that may have influenced firm-level injury outcomes during the study period was not possible.

### Concluding remarks

Findings from the current study show some evidence of effectiveness of the HSEp on firm lost-time injury rate reductions. However, evidence of effectiveness was strongly impacted by COVID-19 pandemic-related injuries. The program effectiveness varied by context, including the time period (time of enrolment, follow-up year), industry sector, firm size, level of participation in the program, and suggests less effectiveness in COVID-19 injury management. While the observed injury rate reductions may be attributed to the safety program, they may also reflect greater organizational capacity or more robust implementation processes in certain firms, though further research is needed to explore these potential mechanisms.

## Supplementary material

Supplementary material
