# Supplementary material for: Evaluating a new voluntary occupational health and safety management system program in the context of a pandemic
Source: Scand J Work Environ Health. 2026 Jun 26;52(4):371–80. doi: 10.5271/sjweh.4285 (PMC13344106; doi:10.5271/sjweh.4285)
Supplement: Supplementary material [file SJWEH-52-371-S001.pdf]

Evaluating a new voluntary occupational health and safety management system program in the context of a pandemic[1]

by Laksika B Sivaraj, PhD, Robert A Macpherson, PhD, Christopher B McLeod, PhD [2]

1. Supplementary material
2. Correspondence to: Dr. Christopher B. McLeod, Partnership for Work, Health and Safety, School of Population and Public Health, University of British Columbia, 2206 East Mall, Vancouver, BC, V6T 1Z3, Canada. [E-mail: chris.mcleod@ubc.ca].

Table S1. Cohort inclusion/exclusion criteria

	**Year 1 participants**		**Year 2 participants**		**Total**	
**Exclusion criteria**	**Cases**	**Controls**	**Cases**	**Controls**	**Cases**	**Controls**
None	1405	452 189	846	452 748	2251	904 937
Firms with no firm data	1388	323 610	840	335 939	2228	659 549
Control to Treatment crossover firms	1388	320 242	840	331 874	2228	652 116
Schedule-2/self-insured firms	1386	319 648	839	331 279	2225	650 927
Firms with <1 FTE for the pre-intervention years	1311	139 684	781	138 852	2092	278 536
Firms with <=0 FTE for the 1st post-intervention year	1302	133 492	779	132 748	2081	266 240
Firms with missing sector/region information	1302	133 487	779	132 744	2081	266 231

Notes: The term "FTE" refers to full-time equivalent.

Table S2. Detailed description of variables used for matching

Variable	Description
Firm size	Firms were categorized based on the FTE workers employed by the firm (FTE <20, 20 to <50, 50 to <100, 100 to <500, >=500) in the baseline year.
NAICS sub-sector (3-digit level)	The baseline NAICS sector was defined at the 3-digit level based on the predominant class.
NAICS group (1-digit level)	Baseline NAICS sectors were grouped based on their first NAICS digit (NAICS-1xx, -2xx, -3xx, -4xx, -5xx, -6xx, -7xx/8xx/9xx).
Safety Groups experience	Since Safety Groups ended in 2019, participation for all cohorts was assessed based on the years 2017 to 2019. Firms with participation for at least one year during this period were classified as having some Safety Groups experience. All others were considered to have no experience.
COR status	Firms that are COR-certified or registered on or before the baseline year, dated back to 2000, were identified as COR-associated.
Pre-intervention lost-time injury history	Firms were classified as having no, one or more lost-time claims in the 3-year pre-intervention period

Notes: "NAICS" refers to the North American Industrial Classification System. "FTE" refers to full-time equivalent. "COR" refer to the Certificate of Recognition program.

Table S3. Pre- and Post-matching baseline lost-time injury rates per 10 000 FTEs

	**Year 1 participants**				**Year 2 participants**			
	Pre-match (N=1302) mean (Q1-Q3)		Post-match (N=1056) mean (Q1-Q3)		Pre-match (N=779) mean (Q1-Q3)		Post-match (N=756) mean (Q1-Q3)	
	Cases	Controls	Cases	Controls	Cases	Controls	Cases	Controls
**Overall sample**	11.35 (0-0)	8.27 (0-0)	11.72 (0-0)	12.00 (0-0)	12.16 (0-0)	8.11 (0-0)	12.10 (0-0)	13.72 (0-0)
**Stratified by firm size**								
<20 FTE	13.74 (0-0)	7.98 (0-0)	15.56 (0-0)	19.67 (0-0)	13.30 (0-0)	7.78 (0-0)	12.71 (0-0)	24.72 (0-0)
20 to <50 FTE	10.98 (0-0)	9.26 (0-0)	11.80 (0-0)	10.87 (0-0)	13.18 (0-0)	9.38 (0-0)	13.26 (0-0)	12.57(0-0)
50 to <100 FTE	13.63 (0-0)	10.29 (0-0)	13.20 (0-0)	11.71 (0-0)	15.84 (0-0)	10.35 (0-0)	15.84 (0-0)	14.33 (0-0)
100 to <500 FTE	10.92 (0-0)	9.45 (0-0)	10.44 (0-0)	10.59 (0-0)	10.51 (0-0)	9.98 (0-0)	10.80 (0-0)	10.10 (0-0)
>=500 FTE	6.88 (0-11.47)	6.30 (0-9.10)	7.86 (0-12.41)	8.47 (0-12.09)	8.75 (0-13.30)	6.12 (0-8.63)	7.72 (0-12.84)	7.33 (0-11.14)

Notes: The term "FTE" refers to full-time equivalent.

Table S4. Intervention effects of HSEp participation on injury rates by Safety Groups experience

		Lost-time claim rates		Non-COVID lost-time claim rates	
	Follow-up	IRR (95%CI)	p-value	IRR (95%CI)	p-value
**Some experience**	*Combined*	1.02 (0.88-1.17)	0.829	0.93 (0.85-1.02)	0.118
	Year 1	1.13 (0.91-1.40)	0.278	0.95 (0.86-1.06)	0.375
	Year 2	0.90 (0.76-1.07)	0.225	0.90 (0.80-1.01)	0.062
	Year 3	1.05 (0.89-1.25)	0.554	0.94 (0.84-1.05)	0.248
	Year 4	1.00 (0.85-1.17)	0.974	0.93 (0.81-1.07)	0.302
**No experience**	*Combined*	0.91 (0.80-1.04)	0.156	0.89 (0.80-1.01)	0.063
	Year 1	1.00 (0.82-1.21)	0.978	0.96 (0.85-1.08)	0.520
	Year 2	0.90 (0.77-1.07)	0.226	0.91 (0.79-1.05)	0.186
	Year 3	0.90 (0.75-1.07)	0.238	0.89 (0.75-1.05)	0.173
	Year 4	0.84 (0.71-0.99)	**0.038**	0.83 (0.71-0.97)	**0.023**

Notes: Estimates derived from matched adjusted population-averaged negative binomial regression models. IRR refers to the incidence rate ratio, and confidence levels are based on a 95% significance level.

Table S5. Type III test results for the covariates and the Quasi-likelihood Information Criterion (QIC) for preferred models of the main analyses

		Lost-time injury rates				Non-COVID lost-time injury rates			
		DF	ChiSq Statistic	Pr > ChiSq	QIC	DF	ChiSq Statistic	Pr > ChiSq	QIC
**Combined follow-up analysis**									
**Year 1 participants**					29 593.89				64 427.93
	HSEp	1	0.11	0.742		1	0.93	0.334	
	Follow-up	1	102.65	**<0.0001**		1	0.63	0.428	
	Calendar year	.	.	.		8	84.38	**<0.0001**	
	Calendar month	.	.	.		11	196.10	**<0.0001**	
**Year 2 participants**					7707.97				27 344.29
	HSEp	1	0.41	0.522		1	0.28	0.598	
	Follow-up	1	90.98	**<0.0001**		1	0.39	0.532	
	Calendar year	.	.	.		8	56.78	**<0.0001**	
	Calendar month	.	.	.		11	88.55	**<0.0001**	
**Construction**					20 162.89				20 398.87
	HSEp	1	0.47	0.495		1	0.67	0.412	
	Follow-up	1	2.64	0.104		1	0.72	0.395	
	Calendar year	.	.	.		8	18.10	**0.021**	
	Calendar month	.	.	.		11	45.72	**<0.0001**	
**Manufacturing**					22 241.73				33 992.67
	HSEp	1	0.07	0.789		1	0.00	0.965	
	Follow-up	1	10.02	**0.002**		1	4.43	**0.035**	
**Healthcare**					-8416.93				6917.97
	HSEp	1	2.02	0.155		1	0.98	0.322	
	Follow-up	1	0.03	0.867		1	6.46	**0.011**	
	Calendar year	8	203.56	**<0.0001**		.	.	.	
	Calendar month	11	225.40	**<0.0001**		.	.	.	
	Firm size	4	99.53	**<0.0001**		.	.	.	
	Region	3	10.46	**0.015**		.	.	.	
**Services**					11 271.41				19 326.91
	HSEp	1	0.02	0.877		1	0.07	0.794	
	Follow-up	1	7.19	**0.007**		1	2.61	0.106	
	Calendar year	8	63.42	**<0.0001**		8	90.25	**<0.0001**	
	Calendar month	11	97.32	**<0.0001**		11	132.45	**<0.0001**	
**Small and medium firms (<100 FTE)**					37 528.84				47 742.38
	HSEp	1	0.31	0.578		1	1.24	0.265	
	Follow-up	1	9.17	**0.003**		1	1.06	0.303	
	Calendar year	.	.	.		8	21.38	**0.006**	
	Calendar month	.	.	.		11	65.47	**<0.0001**	
**Large firms (100+ FTE)**					11 476.91				49 522.42

HSEp	1	0.25	0.620		1	0.83	0.362	
Follow-up	1	137.44	**<0.0001**		1	0.00	0.965	
Calendar year	.	.	.		8	97.91	**<0.0001**	
Calendar month	.	.	.		11	226.46	**<0.0001**	
Region	.	.	.		3	2.28	0.516	
Industry	.	.	.		20	426.83	**<0.0001**	
**Longitudinal follow-up analysis**								
**Year 1 participants**				33 946.83				66 901.62
HSEp	1	0.26	0.613		1	0.93	0.334	
Follow-up year	5	154.26	**<0.0001**		5	89.90	**<0.0001**	
**Year 2 participants**				10 689.03				31 188.01
HSEp	1	0.51	0.477		1	0.29	0.593	
Follow-up year	5	132.05	**<0.0001**		5	41.16	**<0.0001**	
**Construction**				20 306.16				20 469.99
HSEp	1	0.57	0.452		1	1.10	0.294	
Follow-up year	4	1.36	0.852		4	4.47	0.347	
Calendar year	8	20.83	**0.008**		.	.	.	
Calendar month	11	32.26	**0.001**		.	.	.	
**Manufacturing**				23 751.77				36 933.78
HSEp	1	0.26	0.611		1	0.37	0.545	
Follow-up year	4	43.73	**<0.0001**		4	1.99	0.738	
Calendar year	.	.	.		8	37.86	**<0.0001**	
Calendar month	.	.	.		11	97.09	**<0.0001**	
**Healthcare**				-8414.74				6953.88
HSEp	1	3.70	0.054		1	1.76	0.184	
Follow-up year	4	41.51	**<0.0001**		4	42.29	**<0.0001**	
Calendar year	8	221.98	**<0.0001**		.	.	.	
Calendar month	11	181.13	**<0.0001**		.	.	.	
Firm size	4	100.57	**<0.0001**		.	.	.	
Region	3	10.27	**0.016**		.	.	.	
**Services**				11 240.56				19 298.39
HSEp	1	0.19	0.660		1	0.32	0.574	
Follow-up year	4	12.76	**0.013**		4	22.34	**0.000**	
Calendar year	8	49.14	**<0.0001**		8	57.66	**<0.0001**	
Calendar month	11	92.04	**<0.0001**		11	132.32	**<0.0001**	
**Small and medium firms (<100 FTE)**				37 901.59				50 093.28
HSEp	1	0.02	0.877		1	0.91	0.340	
Follow-up year	4	17.53	**0.002**		4	5.62	0.229	
Calendar year	.	.	.		8	6.96	0.541	
Calendar month	.	.	.		11	65.64	**<0.0001**	

Region	.	.	.		3	18.25	**0.000**	
Industry	.	.	.		19	121.17	**<0.0001**	
**Large firms (100+ FTE)**				11 839.22				49 454.06
HSEp	1	0.17	0.677		1	1.51	0.219	
Follow-up year	4	211.83	**<0.0001**		4	16.16	**0.003**	
Calendar year	.	.	.		8	55.78	**<0.0001**	
Calendar month	.	.	.		11	212.86	**<0.0001**	
Region	.	.	.		3	2.28	0.517	
Industry	.	.	.		20	425.22	**<0.0001**	

Notes: The term “DF” refers to the degrees of freedom. “Pr > ChiSq” provides the probability of obtaining a greater Chi-square statistic than that observed, assuming the null hypothesis is true.

Table S6. Intervention effects of HSEp participation on injury rates after excluding healthcare firms

		Lost-time injury rates	
**Stratification**	Follow-up	IRR (95%CI)	p-value
**By cohort**			
**Year 1 participants**	*Combined*	0.88 (0.78-0.99)	**0.037**
	Year 1	1 (0.83-1.19)	0.957
	Year 2	0.84 (0.71-0.98)	**0.031**
	Year 3	0.95 (0.8-1.12)	0.509
	Year 4	0.75 (0.65-0.87)	**<0.001**
**Year 2 participants**	*Combined*	0.92 (0.79-1.07)	0.287
	Year 1	1 (0.77-1.31)	0.994
	Year 2	0.91 (0.77-1.07)	0.264
	Year 3	0.83 (0.72-0.96)	**0.015**
**By firm size**			
**Small and medium**	*Combined*	0.94 (0.79-1.12)	0.510
	Year 1	0.98 (0.72-1.34)	0.912
	Year 2	0.92 (0.72-1.18)	0.530
	Year 3	0.97 (0.8-1.18)	0.771
	Year 4	0.89 (0.72-1.09)	0.257
**Large**	*Combined*	0.87 (0.78-0.98)	**0.027**
	Year 1	1.01 (0.82-1.23)	0.952
	Year 2	0.85 (0.74-0.97)	**0.020**
	Year 3	0.88 (0.76-1.02)	0.096
	Year 4	0.77 (0.67-0.88)	**<0.001**
**By Safety Groups**			
**Some experience**	*Combined*	0.88 (0.77-1.01)	0.076
	Year 1	0.99 (0.78-1.27)	0.956
	Year 2	0.83 (0.71-0.98)	**0.031**
	Year 3	0.91 (0.77-1.07)	0.247
	Year 4	0.8 (0.69-0.93)	**0.003**
**No experience**	*Combined*	0.9 (0.78-1.04)	0.162
	Year 1	1.02 (0.83-1.26)	0.820
	Year 2	0.92 (0.76-1.1)	0.355
	Year 3	0.89 (0.73-1.08)	0.233
	Year 4	0.78 (0.65-0.94)	**0.008**

Notes: Estimates derived from matched adjusted population-averaged negative binomial regression models. IRR refers to the incidence rate ratio. Lower and upper confidence levels (LCL and UCL) are based on a 95% significance level. “Small and medium” refers to firms with fewer than 100 FTE, whereas “Large” refers to firms with 100+ FTE.

Table S7. Intervention effects of HSEp progression on injury rates

		Lost-time injury rates		Non-COVID-19 lost-time injury rates	
	Follow-up	IRR (95%CI)	p-value	IRR (95%CI)	p-value
**No completed action plans**	*Combined*	0.91 (0.79-1.06)	0.232	0.94 (0.84-1.05)	0.273
	Year 1	0.93 (0.73-1.18)	0.544	1 (0.87-1.15)	0.985
	Year 2	0.86 (0.71-1.05)	0.132	0.91 (0.79-1.05)	0.188
	Year 3	0.85 (0.7-1.02)	0.083	0.89 (0.76-1.05)	0.159
	Year 4	1.01 (0.83-1.21)	0.979	0.95 (0.8-1.13)	0.574
**1+ completed action plans**	*Combined*	1.02 (0.9-1.15)	0.791	0.91 (0.83-0.98)	**0.018**
	Year 1	1.16 (0.96-1.42)	0.131	0.93 (0.85-1.02)	0.137
	Year 2	0.92 (0.78-1.08)	0.294	0.89 (0.8-0.99)	**0.039**
	Year 3	1.08 (0.92-1.26)	0.362	0.93 (0.83-1.04)	0.190
	Year 4	0.91 (0.79-1.05)	0.218	0.86 (0.76-0.98)	**0.019**

Notes: Estimates derived from matched adjusted population-averaged negative binomial regression models. IRR refers to the incidence rate ratio. Lower and upper confidence levels (LCL and UCL) are based on a 95% significance level.
